# Physical function assessment tools in pediatric rheumatology

**DOI:** 10.1186/1546-0096-6-9

**Published:** 2008-06-04

**Authors:** Lakshmi Nandini Moorthy, Margaret GE Peterson, Melanie J Harrison, Karen B Onel, Thomas JA Lehman

**Affiliations:** 1 Robert Wood Johnson Medical School-UMDNJ, Dept. of Pediatrics, Division of Rheumatology, New Brunswick, NJ 08903, USA; 2 Hospital For Special Surgery, 535 East 70th Street, New York, NY 10021, USA; 3 Wyeth Research, 500 Arcola Road, Collegeville, PA 19426, USA; 4 Pediatric Rheumatology, The Univeristy of Chicago Medical Center, MC5044, 5841 South Maryland Avenue, Chicago, IL 60637, USA; 5 Pediatric Rheumatology, Hospital for Special Surgery, 535 East 70th Street, New York, NY 10021, USA

## Abstract

Pediatric rheumatic diseases with predominant musculoskeletal involvement such as juvenile idiopathic arthritis (JIA) and juvenile dermatomyositis(JDM) can cause considerable physical functional impairment and significantly affect the children's quality of life (QOL). Physical function, QOL, health-related QOL (HRQOL) and health status are personal constructs used as outcomes to estimate the impact of these diseases and often used as proxies for each other. The chronic, fluctuating nature of these diseases differs within and between patients, and complicates the measurement of these outcomes. In children, their growing needs and expectations, limited use of age-specific questionnaires, and the use of proxy respondents further influences this evaluation. This article will briefly review the different constructs inclusive of and related to physical function, and the scales used for measuring them. An understanding of these instruments will enable assessment of functional outcome in clinical studies of children with rheumatic diseases, measure the impact of the disease and treatments on their lives, and guide us in formulating appropriate interventions.

## Introduction

Pediatric rheumatic diseases causing arthritis, fatigue, muscle weakness and blindness are associated with significant functional impairment. For several children with rheumatic illness, physical functional ability is often the chief determinant of their well-being. Quality of life (QOL), health-related QOL (HRQOL), physical function and health status scales are all used as outcome measures in children with significant musculoskeletal involvement, and account for varying degrees of patient-perceived state of physical ability/and or impact of physical disability on overall well being.

The World Health Organization QOL group defined QOL as "individuals' perceptions of their position in life in the context of the culture and value systems in which they live and in relation to their goals, expectations, standards and concerns. It is a broad ranging concept affected in a complex way by the person's physical health, psychological state, level of independence, social relationships, and their relationships to salient features of their environment" [1]. Calman et al has presented a "goal-oriented" model where QOL measures the difference between a person's expectations and current position at a certain time[2]. Recent scientific advances have increased life span, often at the expense of increased medication use and commensurate drug-related complications, more frequent medical visits and evaluation, all of which can lead to significant emotional and lifestyle changes. QOL emerges as a critical issue in this regard and becomes a primary consideration for the improvement in modern medicine.

Specifically for patients with chronic diseases, health primarily impacts the overall QOL. HRQOL is defined as "optimum levels of mental, physical, role and social functioning, including relationships, and perceptions of health, fitness, life satisfaction and well-being" [3]. Implicit in HRQOL is the "assessment of patient's satisfaction with treatment, outcome and health status and with future prospects" [3]. Health status usually refers to general physical and mental health and is often weighted towards symptoms and physical function. Sometimes, disease-specific measures of impact on organ systems are used to denote health status. In clinical studies, there is considerable overlap between QOL, HRQOL and health status. Since QOL, HRQOL, and health status are distinct constructs measuring different patient-specific information, all three deserve appropriate consideration in clinical outcome studies [4]. Scales used for assessment of physical function and HRQOL in pediatric rheumatic diseases have been reviewed previously [5-11]. In the following sections, we will provide an updated review of tools used to assess physical function measures.

Pediatric rheumatic diseases comprise a heterogeneous group of diagnoses that have different clinical features, complications and prognosis such as juvenile idiopathic arthritis (JIA), dermatomyositis, systemic lupus erythematosus (SLE), vasculitis, and scleroderma. Physical ability is a very relevant outcome in children with predominantly musculoskeletal involvement such as JIA and dermatomyositis [12-17]. We will briefly discuss the impairment of physical function in JIA and dermatomyositis and in all the other pediatric rheumatic diseases; problems encountered in measuring outcomes such as physical function and HRQOL; and the different scales used in practice.

### Juvenile Idiopathic Arthritis

Juvenile Idiopathic Arthritis (JIA) is a term comprising subtypes of chronic inflammatory arthritides, causing erosive arthritis in children, often progressing to disability. These children experience functional impairment due to joint and back pain, heel pain, swelling of joints and morning stiffness, contractures, pain, and anterior uveitis leading to blindness. Children with rapidly progressive destructive synovitis, early involvement of small joints of hands and feet, unrelenting inflammation, subcutaneous nodules, and rheumatoid factor positivity are at significant risk for long-term disability [18]. Many children develop severe erosive hip disease along with other joint involvement, resulting in contractures, joint destruction and ankylosis, often necessitating joint replacements. Children with long-standing disease may have significant growth retardation including short stature and failure to thrive.

Severe erosive disease can cause significant loss of joint motion, an important determinant of functional disability in children [19]. Depending upon the severity and extent of disease involvement, children often experience significant pain and disability, which impede the execution of ordinary activities. [20].

Oliveira et al conducted a multinational, multicenter, cross-sectional study of patients with JIA and assessed proxy-reported HRQOL with the Child Health Questionnaire (CHQ) and compared with that of age-matched healthy children from the same geographic area. Over 6,000 participants were enrolled from 32 countries. The physical and psychosocial summary scores of the CHQ were significantly lower in children with JIA compared to healthy children with greatest impairment in the physical well-being domain. Patients with persistent oligoarthritis had better HRQOL compared with other subtypes, whereas HRQOL was similar across patients with systemic arthritis, polyarthritis, and extended oligoarthritis. Physical wellbeing was influenced by the level of functional impairment, and psychosocial health by the intensity of pain [21].

Shaw et al examined HRQOL of 308 adolescents with juvenile idiopathic arthritis (JIA) using the Juvenile Arthritis Quality of Life Questionnaire (JAQQ) in UK. HRQOL of adolescents with JIA was less than optimal, particularly in the domains of gross motor and systemic functioning. Items most frequently rated as adolescents' biggest psychological problems were "felt frustrated" and "felt depressed," thus confirming the widespread effects of JIA [22].

Since JIA has such widespread impact on physical function, functional ability has been included as one of the core set of outcome variables for the assessment of children with JIA and these core response variables are also valuable in defining flare in them [23,24]. Aggressive treatment along with physical and occupational therapy remain the keystones of managing severe disease.

### Dermatomyositis

Dermatomyositis is a rare chronic inflammatory disease causing significant weakness of the proximal muscles associated with characteristic skin rash. Muscle involvement causing motor weakness hinders children from performing routine activities such as walking, getting up from bed, combing hair and eating food. Generalized fatigue, fever and muscle pain and weakness could worsen functional status. Children with persistent inflammatory disease may exhibit diminished muscle mass due to atrophy, calcific nodules and flexion contractions that cause substantial functional impairment. Physical function is a core set-outcome criterion for measuring clinical outcome and assessing clinical improvement in children with myositis [25]. Functional ability and muscle strength assessments were designated for both activity and damage core sets [26].

### Other rheumatic diseases and medication related morbidity

Other inflammatory vasculitic diseases cause functional impairment due to fatigue, arthritis, myositis, rash, and organ damage. In some instances these may be disfiguring and lead to blindness, paralysis or cognitive impairment. Children with scleroderma may experience skin tightening that is significant enough to cause contractures. In cases of severe morphea, the involvement of the skin may be so profound that it may involve bony tissue, possibly leading to complete non-use of the affected limb.

In addition to the disease-related causes of functional limitation, many children with the systemic lupus erythematosus (SLE) suffer consequences of long-term steroid use, such as avascular necrosis of the joints, severe osteoporosis which makes bones prone to frequent fracturing, requiring surgical joint repair and/or replacement to improve function. Moreover, children on steroids are prone to infection and experience delayed healing of wounds due to their compromised immune status thus complicating their post-operative course. Steroid-related side-effects include obesity, cushingoid features, acne and growth retardation leading to low self-esteem in children that in addition to the disease-related morbidity may reduce their motivation to participate in physical activities.

For children with rheumatic disease, functional impairment encompasses physical, visual and cognitive limitation, and is complicated by the psychosocial impact of having a chronic disease. As pain and fatigue significantly influence children's well being, these should be critical considerations in the measurement of physical ability.

Children with pain amplification syndrome can experience severe functional limitations requiring intensive physical therapy often combined with counseling.

## Problems encountered while assessing outcomes in children

The heterogeneity of rheumatic diseases causing functional impairment, children's changing cognitive skills, needs and expectations, limited availability of questionnaires during the transition from childhood to adulthood, overlap in the constructs measured by these scales, as well as the use of proxy respondents are all factors that complicate measurement of physical function, health status, QOL and HRQOL in children [27-31]. Many instruments modified from adult scales may not take into account a specific phase of children's development which impacts cognitive function, autonomy, body image, expectations, level of independence and recall [32]. Age and language-adjusted formats with items relevant for each age group are ideal for the evaluation of physical function and related outcomes in children over a broad age-range. As most rheumatic diseases are chronic, frequently progressing into adulthood, it is essential to address the lack of instruments to measure physical function, QOL, HRQOL and health status through this phase of growth. Appropriate transitional measures can enable prospective follow-up from childhood to adulthood and facilitate the formulation of both clinically and methodologically accurate comparisons. The Childhood Health Assessment Questionnaire (CHAQ) and the Health Assessment Questionnaire (HAQ) can be used in child and adult populations to measure physical function and enable evaluation during the transition process.

The level of parent-child agreement in children with arthritis and other conditions appears to vary for disability, pain, QOL, and HRQOL [28,33-35]. Waters et al examined parent and adolescent agreement on physical, emotional, mental and social health and well-being in a representative population using the Child Health Questionnaire. Authors reported that adolescents were much less positive about their health and well-being compared to their parents, and were only in close agreement on aspects of health and well-being they rated highly [36]. Greater correlation between child and parent reports has been noted in the more objective domains such as physical function as compared to the social and emotional domains. Brunner et al examined agreement 58 child-parent dyads from Rheumatology clinics and found good agreement for scores of the Childhood Health Assessment Questionnaire (CHAQ), Juvenile Arthritis Quality of life Questionnaire (JAQQ) and moderate agreement for Pediatric Quality of Life inventory (PedsQL), visual analog scale of wellbeing, and the standard gamble utilities [37]. Wagner et al explored the function of children's illness-related cognitive assessments in the parent-child adjustment relationships specific to the context of juvenile arthritis, and found that increased parental distress and child illness intrusiveness were related to increased depressive symptoms in the child [38].

Palmisani et al investigated concordance between physicians and parents in rating the degree of functional ability of children with juvenile idiopathic arthritis (JIA). Concordance, parent over-rating and physician over-rating were observed in 107 (69%), 29 (18.7%) and 19 (12.3%) evaluations, respectively. Parent over-rating was associated with greater intensity of pain (p = 0.01) and higher Childhood Health Assessment Questionnaire (CHAQ) score (p = 0.004), whereas physician over-rating was associated with more severe joint disease (p = 0.04 to <0.001), higher C-reactive protein (p = 0.03) higher frequency of Steinbrocker functional class = II (p < 0.001), and greater articular damage, as measured with the Juvenile Arthritis Damage Index (p < 0.001). Overall, the physicians and parents revealed fair concordance in rating functional ability of children with JIA [39].

Shaw et al found acceptable agreement for pain, general well-being, functional disability, and HRQOL among 303 adolescents with JIA and parents Where discrepancies occurred, parents rated functional ability worse than did adolescents. Although proxy report is likely to be valid for adolescents with JIA at either mild or severe end of the spectrum and/or for the visible manifestations of the disease, there was a wide variation in agreement between adolescents with JIA and their parents that is dependent on the type of health-related variable [40].

These findings stress the need to explore the relationship between the child and parent factors in child adjustment. The level of child-parent agreement varies with child's physical and health status, parent's physical and emotional well being, domain assessed, instrument used, and the construct measured. Obtaining child-reports in conjunction with parent-reports and further exploratory studies will contribute to the constructive examination of the determinants of concordance.

Other potential limitations of the functional status measures include ceiling effect, score inflation by inflammatory pain, reversibility of functional limitations (when secondary to inflammation instead of true damage), non applicability in younger children, lack of clinical studies to establish all the psychometric properties, and increased length and problems with administering them (such as the need for skilled personnel). Further, most of these physical function scales measure functional ability and in some cases general health status (such as the Short Form-36 and the Child Health Questionnaire) but cannot be used as proxies for HRQOL or QOL. Table 1 lists the different scales under categories of physical function, health status, HRQOL and QOL.

**Table 1:** Classification of assessment tools in pediatric rheumatology

**Physical Function**
1. Childhood Health Assessment Questionnaire (CHAQ)
2. Juvenile Arthritis Functional Assessment Scale (JAFAS)
3. Juvenile Arthritis Functional Assessment Report (JAFAR)
4. Juvenile Arthritis Functional Status Index (JASI)
5. Juvenile Arthritis Functionality Scale (JAFS)
6. Juvenile Arthritis Foot disability Index (JAFI)
7. Child Activity Limitations Interview (CALI)
8. Childhood Arthritis Impact Measurement Scales (CHAIMS)
9. Functional Status Measure FSII (R)
10. Steinbrocker Classification
11. Weighting of joint counts
12. Childhood Myositis Assessment Scale (CMAS)
Health Status*
1. Short-Form General Health Survey SF-36 and Short Form-20 (SF-20)
2. Child Health Questionnaire (CHQ)
3. Childhood Arthritis Health Profile (CAHP)
4. The Child Health and Illness Profile chip (CHIP)

Health-related QOL and QOL*
1. Pediatric QOL Inventory (PedsQL) – generic and rheumatology module
2. Juvenile Arthritis Quality of Life Questionnaire (JAQQ)
3. EQ5D (EuroQOL)
4. TNO AZL Children's Quality of Life questionnaire (TACQOL)
5. Quality of My Life Visual Analog Scale
6. Simple Measure of Impact of Lupus Erythematosus in Youngsters^© ^(SMILEY^©^)

This table lists some of the commonly used scales used to assess physical function, health status and QOL.

*Sometimes health status, HRQOL and QOL scales are used interchangeably

## Commonly used outcome measures in pediatric rheumatic diseases

Pediatric studies typically use descriptive health status scales, which explore various domains and their relationship with each other, instead of utilities or preference-based measures, which quantitatively assess individual preferences. With utility scales, results have been variable in case of children with musculoskeletal diseases, and therefore the account will focus on descriptive measures [41]. The choice of the instrument is dependent upon the extent of the impact and type of pediatric rheumatic disease, since there are some scales that focus on measuring the impact of physical function alone and others that assess physical function as a part of the assessment of a more global construct such as health status or QOL. Although these scales are most often used in JIA, they have a role in characterizing physical function in children with lupus, scleroderma, and pain amplification syndromes.

### Childhood Health Assessment Questionnaire (CHAQ)

The Childhood Health Assessment Questionnaire (CHAQ), a widely used valid and reliable measure of physical function in children with rheumatic diseases is adapted from the Health Assessment Questionnaire (HAQ) [17,42-44]. The HAQ, used widely in adults, takes into account drug side effects, death and dollar costs in addition to disability, discomfort and pain, although the commonly used components for outcome studies are the disability index, discomfort and pain scales. The CHAQ consists of both child- (8–19 years) and parent-reports (2–19 years), takes less than 10 minutes to complete, is easy to administer, score and interpret, with main areas of focus being disability and discomfort. The CHAQ domains estimating disability index and the pain scales and scoring are analogous to those in the HAQ. After 19 years of age, the HAQ can be used to assess physical function. The CHAQ does not take into account drug side effects, death and dollar costs. The 30 items relating to disability pertain to problems or limitations in the past week attributable to illness in the eight domains of dressing and grooming, arising, eating, walking, hygiene, reach, grip, and activities. The answers are in the form of a 4-point Likert scale with an additional option "not applicable." Disability index, estimated as the mean of the eight domain scores ranges from 0–3, where zero indicates no disability and three translates to severe disability. The score accounts for the aids or devices used, and assistance needed to perform the listed activities. Doubly anchored visual analog scales evaluate Discomfort and Global assessment [17].

When originally developed in 1994, the CHAQ was found to have excellent psychometric properties including test-retest reliability, convergent validity, and good correlations with other scales measuring related constructs such as the Steinbrocker's functional class, active joint count, disease activity and degree of morning stiffness and subsequently has been found to be responsive [17,45]. Ruperto et al compared the relative responsiveness of outcome measures in 26 children with oligoarticular juvenile chronic arthritis which included physician and parent global assessments, functional ability as assessed by the CHAQ, articular variables, and laboratory markers of systemic inflammation. These outcomes were assessed at admission and 3 months later. Standardized response median, the effect size, and the Guyatt methods were used to calculate responsiveness. The most responsive measures were the physician global assessment of disease activity, and articular variables such as the active joint count, global articular severity score, and the number and score of swollen joints. The parent global assessment of the child's well being, the scores of joints with pain/tenderness and limited range of motion, and the number of joints with limited range of motion displayed intermediate responsiveness. Among the least responsive measures were the CHAQ, morning stiffness, and laboratory indicators of systemic inflammation [46].

The minimal clinically important difference (MCID) has been calculated for the CHAQ scores to follow long-term outcomes in children with JIA [47]. Brunner et al calculated changes in CHAQ scores for patient ratings (n = 67) between clinic visits. Changes in patient well-being, disease activity and the occurrence of flare or important improvement between visits were used as standards for the MCID. MCIDs were defined as the median changes of the CHAQ scores of individual patients who had a minimal important improvement or worsening between visits. The median change in CHAQ scores of patients who rated themselves or were rated by others as unchanged was often 0. Depending on the external standard used, the maximum MCID was -0.188 for improvement and +0.125 for worsening. Authors found that the minimal clinically important differences (MCID) of the CHAQ for both worsening and improvement are often close to the level of the smallest potential difference (0.125), suggesting that the CHAQ may be relatively insensitive to short term changes in children with JIA [48].

Rasch analysis was used to to compare the difficulty of each of the 30 items for children of 2 age groups (> or = 10 years old and <10 years old). Although, 8 of the 30 items (27%) of the CHAQ were rated significantly different in the 2 age groups, the impact on the CHAQ disability index using its original scoring system remained low (about 0.25 points on a scale of 0–3). CHAQ's design and scoring system appeared to remove the bias due to difference in physical development [49].

The CHAQ has been extensively used in assessing functional outcome in children with arthritis and has been translated into several languages and cross-culturally adapted in a number of countries[50]. It has been used to measure physical function in children with SLE [51]. CHAQ is also valid, internally reliable (inter-item correlation range 0.35–0.81, n = 115), and responsive (effect size = 1.05 and standardized response mean = 1.20) in assessing physical function in children with idiopathic inflammatory myositis and is used as an important measure of functional outcome in clinical trials [52].

### Juvenile Arthritis Functional Assessment Scale (JAFAS) and Juvenile Arthritis Functional Assessment Report (JAFAR)

The Juvenile Arthritis Functional Assessment Scale (JAFAS) assesses physical function in children aged 7–16 years in clinic settings. It is a comprehensive measure of physical function, which requires standardized equipment and entails administration by a trained health professional, physical or an occupational therapist, who times the child's performance on 10 tasks [15]. The JAFAS is reliable and valid, but its responsiveness has not been established. It has been used along with the CHAQ as a measure of functional ability in a study examining the relationship between joint impairment and physical function [19]. The JAFAS has been used as a standard measure of physical function in a study to evaluate the reliability and validity of a Spanish version of the CHAQ [53].

Bekkering et al conducted a cross-sectional study in 28 children with JIA to compare the measurement properties JAFAS with CHAQ on the level of individual items. Cronbach's alpha was high for both the JAFAS (0.92) and the CHAQ (0.96). The Spearman correlation coefficient between the JAFAS and the CHAQ was significant (0.55, p < 0.01). With six out of ten items, the JAFAS classified the child as less disabled than with corresponding CHAQ activities. Overall, associations with measures of disease activity and joint range of motion were higher for the CHAQ than for the JAFAS [54].

The Juvenile Arthritis Functional Assessment Report (JAFAR), a reliable and valid 23-item scale evaluating physical functional ability has both child- and parent- reports [16]. The responses are in the form of 3-point scales, and total scores range from 0–46, where lower scores indicate better function. Both these versions have good construct validity, reliability and responsiveness [16,55]. The JAFAR has been used in multiple clinical studies to assess physical function in children with JIA [38]. While measuring convergent validity of the Childhood Myositis Assessment Scales (CMAS), JAFAR was one of the standard measures of physical function [56]. The content for both scales JAFAS and the JAFAR was derived from the HAQ, AIMS, and the McMaster Health Index Questionnaire [57]. The greatest limiting feature for both scales is that they cannot be used in children under 7 years of age. Additionally, JAFAS requires a skilled trained professional for test administration.

### Juvenile Arthritis Functional Status Index (JASI)

Juvenile Arthritis Functional Status Index (JASI) evaluates activities of daily living and functional mobility in children with JIA between 8–18 years of age [12,13]. Items for JASI were generated rigorously after interviews of children, parents, teachers, clinicians, and subsequently consolidated on priority basis. JASI part I is composed of 100 functional items divided into 5 groups that include self-care, domestic, mobility, school, and extracurricular. Responses are in the form of 7 point rating Likert scale. JASI Part II is more patient-specific, where the child delineates up to five tasks that are difficult to perform and these are assessed on subsequent follow-up on an individualized basis. The JASI has excellent reliability and construct validity, but its responsiveness has not been established. Despite good measurement properties, its use is limited because of the longer time taken to administer the test (over 40 minutes) and the lack of validity in children less than 8 years of age.

### Juvenile Arthritis Functionality Scale (JAFS)

The Juvenile Arthritis Functionality Scale (JAFS), a new short is a 15-item questionnaire of physical function in children with juvenile idiopathic arthritis (JIA), explores physical function in 3 body areas (lower limbs, hand/wrist, and upper segment). Validation of the Italian version of this scale was carried out by evaluating 211 children with JIA. The JAFS was found to be reliable, internally consistent (Cronbach's alpha = 0.82), valid, responsive (standardized response mean = 0.42 to 0.56), and have discriminative ability [58].

### Juvenile Arthritis Foot disability Index (JAFI)

The juvenile arthritis foot disability index (JAFI) is a new scale derived from the International Classification of Functioning, Disability and Health that included 27 statements classified into the following dimensions: Impairment, Activity Limitation, and Participation Restriction[59]. Thirty-six children/adolescents with JIA and 29 healthy subjects participated. Parents and adolescents were asked to comment on the content. Increasing JAFI scores was found along with increasing joint impairment scores, CHAQ scores, and self-rated foot-related participation restriction. Children with JIA had more prominent foot-related disability as assessed by JAFI compared to healthy controls. Authors found no internal redundancy (r_s _> 0.90) between items. Internal consistency within each subscale was acceptable (r_s _> 0.50) for all items but one. They found good test-retest reliability (weighted kappa coefficients for the 3 JAFI dimensions were 0.90, 0.85, and 0.88). One preliminary examination, authors reported JAFI to be valid and reliable[59].

### Child Activity Limitations Interview (CALI)

The Child Activity Limitations Interview (CALI) was developed in order to measure functional impairment secondary to recurrent pain in school-age children and adolescents, and to compare this measure to the Functional Disability Inventory. Subjects comprised 189 children with mean age 12.4 years, with diagnoses of headaches, juvenile idiopathic arthritis, and sickle cell disease. Internal consistency of the CALI was excellent (alpha = 0.88, child version; alpha = 0.95, parent version). Results showed that CALI had good internal consistency and moderate test-retest reliability and cross-informant reliability. CALI May be useful for assessing and following the subjective report of functional impairment in school-age children and adolescents with recurrent and chronic pain [60].

### Childhood Arthritis Impact Measurement Scales (CHAIMS)

The Childhood Arthritis Impact Measurement Scales (CHAIMS), adapted from Arthritis Impact Measurement Scales (AIMS) used in adults, was the first disease-specific measure developed for children with JIA [14,61]. Despite measuring physical function and discomfort in children, this scale is not widely used [11,14,62].

### Functional Status Measure FSII (R)

The Functional Status measure FSII (R), evaluates health status of children aged 0–16 years with chronic diseases that predominantly affect physical function [63]. The FSII (R) is a validated general health status measure in children between ages of 0 to 16 with 47-item (long form) and 14-item (short form) parent-reports. The FSII (R) addresses the areas of eating, sleeping, play behavior and emotional health, and has been demonstrated to have good convergent validity and internal consistency.

### Steinbrocker classification

Developed over 5 decades ago, the Steinbrocker classification divides patients into four functional classes based on their abilities to perform activities of daily living, and is used to evaluate short- and long-term functional outcome of patients with JIA [64]. There is a wide range of disability included in these classes and strict delineation between subgroups is often not possible. With the advent of newer instruments that integrate evaluation of physical, social and mental domains, this instrument is used less often.

### Weighting of joint counts

A panel of pediatric rheumatologists designed a weighted joint score, where weights were assigned from 1 (not very important) to 10 (essential for key functional activities) to each joint [65]. Weighted counts of swollen and active and/or painful joints had greater correlation as compared to simple counts with the physician's global assessment, parent's assessment of overall well-being and intensity of pain than did simple counts. Weighting increased most of the correlations between joint counts and the CHAQ, score and the physical component of the Child Health Questionnaire (CHQ) [65].

### Childhood Myositis Assessment Scale (CMAS)

The Childhood Myositis Assessment Scale (CMAS) has been designed as a 14-item quantitative functional assessment tool used for evaluating axial and proximal muscle function in the context of strength and endurance in children with idiopathic inflammatory myopathies [56,66]. Takken et al found that CMAS, CHAQ and CHQ correlated with muscle strength and maximal oxygen consumption [67].

## Other scales that measure health status and QOL

The following scales do not solely assess physical function but measure health status and QOL. We have mentioned these scales briefly in this review because physical function is measured as an important domain of the health status, QOL and HRQOL.

### Juvenile Arthritis Quality of Life Questionnaire (JAQQ)

The Juvenile Arthritis Quality of Life Questionnaire (JAQQ) was developed as a valid and responsive disease-specific measure of physical and psychosocial function in children afflicted with JIA, including spondyloarthropathies [9,68-71]. Children and their parents completed a questionnaire addressing physical and psychosocial function, and general symptoms and rated items based on the frequency of occurrence and importance. This method of item-generation ensured content relevance. Subsequently, the experts rated these items for their potential responsiveness and grouped them into four categories including gross motor function, fine motor function, psychosocial function and general symptoms. JAQQ was successfully used to measure HRQOL in adolescents with JIA in UK, but authors concluded that developmentally appropriate issues should be included [22].

### Childhood Arthritis Health Profile (CAHP)

The Childhood Arthritis Health Profile (CAHP) was developed to evaluate the global physical and psychosocial health status of children over 13 years with JIA. It is a parent-report comprising the following domains: physical functional status, psychosocial functioning, behavior, general health perceptions, and family functioning and impact of disease [72,73]. This scale consists of the CHQ as the core general health status measure and additionally consists of a module focused on juvenile arthritis [74,75]. The domains of CAHP are relevant to children with arthritis, but due to lack of sufficient studies published using the CAHP, lack of parallel child-report, and inapplicability in children under 13 years of age limits its use.

### Short-Form General Health Survey SF-36 and Short Form-20 (SF-20)

The Short-Form General Health Survey SF-36 and Short Form-20 (SF-20) are widely used health status measures used in clinical studies of healthy and diseased adults and older adolescents including those with SLE and other rheumatic diseases [76-86]. Being reliable, valid and responsive, the SF-36 consists of multi-item scales that measure eight domains: physical functioning, role limitations due to physical health and emotional problems, bodily pain, social functioning, general mental health covering psychological distress and well-being, vitality, energy or fatigue, and general health perceptions [77,78,87]. The major limitation of studies using SF-36 or SF-20 is that they claim to be measuring QOL when they are actually using an instrument derived to measure health status. Therefore a cautious review of studies using these instruments is critical.

### Child Health Questionnaire (CHQ)

The Child Health Questionnaire (CHQ), adapted form the SF-36, evaluates the child's overall health status through following domains: general health perceptions, physical functioning, general behavior, mental health, emotional or time impact, on the parent, family cohesion, change in health, bodily pain, limitations in school, work, and activities with friends due to physical problems and due to emotional and behavioral difficulties, behavior, mental health, and self-esteem, and limitations in family activities [74]. There are both parent-(above 5 years) and child-reports (above 10 years), but the reports are not parallel. The CHQ has been found to be sensitive to clinical changes in children with JIA [88].

### Pediatric QOL Inventory (PedsQL)

The Pediatric QOL Inventory (PedsQL) 4.0 generic module is a brief, valid and reliable measure of QOL in healthy and sick children between 2–18 years [27,89-91]. PedsQL4.0 has both parent- and child-reports with separate language-adjusted formats for the various age-groups [27]. Both versions comprise of 23 items that address the following four domains: physical, emotional, social, and school functioning. The total, physical and psychosocial summary scores (average of emotional, social and school functioning scores) are calculated on a 0–100 scale, with higher score indicating better QOL. Varni et al showed that the PedsQL appeared to influence clinical decision-making leading to increases in HRQOL in rheumatology clinic, and was sensitive to changes in clinical status over time in the orthopedic clinic [92]. The PedsQL Rheumatology module, designed similar to the generic module has 22 items grouped into five domains of pain and hurt, daily activities, treatment, worry, and communication. Together the generic and rheumatologic modules take about 10–15 minutes to complete and have shown to be reliable, valid, and responsive in pediatric rheumatic diseases.

The Child Health and Illness Profile, largely used for adolescent research, is a valid and reliable general health status measure for children between ages 11 and 17 years [93-98]. The EuroQOL, a generic health utility index extensively used in adult studies, has been shown to have validity in assessing QOL in children with JIA [99]. The TNO AZL Children's Quality of Life questionnaire (TACQOL) is a generic parent- report of HRQOL in children aged 6–15 years [29]. The Quality of My Life Visual Analog Scale has been used for measuring the overall QOL and HRQOL in children [4]. The Simple Measure of Impact of Lupus Erythematosus in Youngsters^© ^(SMILEY^©^), a valid and reliable brief questionnaire with parallel child and parent versions was developed specifically to measure HRQOL in children with SLE [100].

## Conclusion

Physical ability remains very important in children for the development of their motor skills, self-esteem and independence. Therefore, further refinement of existing instruments, and the examination of their relationship with cognitive skills, self-esteem and other markers of development would contribute to a greater understanding of the impact of physical disability in this population.

## Competing interests

LNM: No financial/non-financial competing interests

MGEP: No financial/non-financial competing interests

MJH: Full-time employee of Wyeth Research. All work presented here was conducted prior to Dr. Harrison's current employment and was not supported by Wyeth.

KBO: No financial/non-financial competing interests

TJAL: Speakers Bureau- Abbott, Wyeth, Genentech and Genzyme

## Authors' contributions

LNM is the main author and the corresponding author and has primarily designed and written the article, Co-authors, MGEP, KBO, MJH, and TJAL have made substantial contributions to conception and design; have been involved in revising the manuscript and have given final approval of the version to be published. All authors read and approved the final manuscript.
